# Sustainable intensification of climate-resilient maize–chickpea system in semi-arid tropics through assessing factor productivity

**DOI:** 10.1038/s41598-024-53573-4

**Published:** 2024-02-17

**Authors:** S. R. Salakinkop, S. C. Talekar, C. R. Patil, S. B. Patil, S. L. Jat, K. S. Iliger, G. Manjulatha, S. I. Harlapur, R. M. Kachapur

**Affiliations:** 1 All India Coordinated Maize Improvement Project, Main Agricultural Research Station, University of Agricultural Sciences, Dharwad, Karnataka 580 005 India; 2 Institute of Organic Farming, University of Agricultural Sciences, Dharwad, Karnataka 580 005 India; 3 All India Coordinated Dryland Improvement Project, University of Agricultural Sciences, Dharwad, Karnataka India; 4 Indian Institute of Maize Research, Delhi Unit, Pusa Campus, New Delhi, India; 5 Agriculture Research Station, Karimnagar, 505 001 India

**Keywords:** Ecology, Plant sciences

## Abstract

Global trends show that the rapid increase in maize production is associated more with the expansion of maize growing areas than with rapid increases in yield. This is possible through achieving possible higher productivity through maize production practices intensification to meet the sustainable production. Therefore, a field experiment on “Ecological intensification of climate-resilient maize–chickpea cropping system” was conducted during consecutive three years from 2017–2018 to 2019–2020 at Main Agricultural Research Station, Dharwad, Karnataka, India. Results of three years pooled data revealed that ecological intensification (EI) treatment which comprises of all best management practices resulted in higher grain yield (7560 kg/ha) and stover yield compared to farmers’ practice (FP) and all other treatments which were deficit in one or other crop management practices. Similarly, in the succeeding winter season, significantly higher chickpea yield (797 kg/ha) was recorded in EI. Further EI practice recorded significant amount of soil organic carbon, available nitrogen, phosphorus, potassium, zinc, and iron after completion of third cycle of experimentation (0.60%, 235.3 kg/ha,21.0 kg/ha,363.2 kg/ha,0.52 ppm and 5.2 ppm respectively). Soil enzymatic activity was also improved in EI practice over the years and improvement in each year was significant. Lower input energy use was in FP (17,855.2 MJ/ha). Whereas total output energy produced was the highest in EI practice (220,590 MJ ha^−1^) and lower output energy was recorded in EI–integrated nutrient management (INM) (149,255 MJ/ha). Lower energy productivity was noticed in EI-INM. Lower specific energy was recorded in FP and was followed by EI practice. Whereas higher specific energy was noticed is EI–INM. Each individual year and pooled data showed that EI practice recorded higher net return and benefit–cost ratio. The lower net returns were obtained in EI-integrated weed management (Rs. 51354.7/ha), EI-recommended irrigation management (Rs. 56,015.3/ha), integrated pest management (Rs. 59,569.7/ha) and farmers’ practice (Rs. 67,357.7/ha) which were on par with others.

## Introduction

Maize (*Zea mays* L.) is known as the “Queen of Cereals” and is one of the most extensively planted cereal crops in the world, ranking first in terms of production. Annual global demand for maize, rice, and wheat is predicted to exceed 3.3 billion tons by 2050 and this will have to happen from similar or perhaps much lower land resources^1^. The global average yield for maize has been increasing steadily over the period. Yields have been improving at a rate of 65 kg/ha/year^2^ since 1960 which accounts to steady rate of 10 million ton/year until 2004, after which they shifted to a steeper production of 31 M ton/year^2^. The increase in production closely follows the recent trend for maize area expansion, which had been increasing at a rate of 0.9 million ha/year prior to 2007 and, has now been increasing at the more rapid pace of 4.7 million ha/year. Simultaneously, the shifting climate and environmental degradation, changing climate, and diseases and insect pests are known threats to crop production and productivity, especially in the tropics. The increased output must be achieved with less land, water, energy, and other vital inputs, as well as a limited natural resource base. Agriculture in Asia faces new challenges because of climate change. Climate change is thought to have affected worldwide maize and wheat yields by 3.8% and 5.5%, respectively, since 1980. Climate change has an impact on crop yields as well as food availability and productivity of natural resources including land and water^3,4^. South Asia's natural resources are 3–5 times more stressed than the rest of the world due to demographic, economic, and political pressures^4^^.^

After rice and wheat, it is India's third most important cereal. It accounts for 9.1% of the India’s total whole food grain production. About 20–25% of India's maize is used for human consumption, 60–65% for cattle and poultry feed, and 10–15% for food processing industries such as corn flakes, dextrose, starch, popcorn, corn oil, and corn syrup. The world's maize area is 192.50 million hectares, and it ranks first in production with 1,112.40 million metric tonne^1^. In India, maize is grown in an area of 9.6 m ha with 28.8 m t of production and 3006 kg/ha productivity, while in Karnataka it has 1.6 m ha of area with 4.2 m t of production and 2990 kg ha^−1^ productivity^5^. However, demand for maize in India by end of 2022 is estimated at 44 million metric tons. And not less than 15 million farmers are engaged in maize cultivation in India^5^. During the previous 5 years maize’s annual growth rate was 11% and is sourced for more than 35,000 products^5^.

Despite several constraints, such as overdependence on rainfall, frequent climatic extremes such as drought, heat, and/or waterlogging, yield losses due to pre- and postharvest pathogens and insect pests, weeds, and lack of access to high-quality seed in some areas, several Asian countries have seen impressive growth rates in maize area, production, and productivity in recent years. However, alternative climate-smart, long-term intensification strategies in the tropics must be considered in addition. As a result, developing climate resilience in Asia necessitates a multi-disciplinary approach. More widespread understanding and implementation of climate-smart agronomic management practices, as well as the enhancement of local competencies and an emphasis on sustainability, are all part of this strategy. Maize is primarily grown as rainfed crops by smallholder farmers in most of Asia.

Yield and yield components of corn were significantly affected by planting patterns, plant densities and maize hybrids^6^. Corn hybrids respond differently to high plant density^7^. Several researchers reported that the effects of row spacing and hybrids on maize dry matter yield and quality characteristics are variable^7,8^. Pest infestations can also be influenced by plant density or row spacing. Root maggots were minimized when the plant density was high (*Delia* spp.). On the contrary, high density reduced grain yield due to increased pollen to silking interval resulting in more barrenness of ears. However, it may lead to higher risk of lodging, hence causing significant yield loss of the crop^9^. Regional environmental condition is the key factor for determining the planting density, and reasonable cultivation techniques and appropriate density-resistant varieties are effective approaches to overcome environmental constraints and increase planting density^10^.

Due to moderate organic matter oxidation, minimum soil cultivation restricts respiration gases in the rooting zone, porosity for water transport, retention, and release, and inhibits the re-exposure of weed seeds and their germination^11^. Irrigation is another key management strategy for increasing crop output and improving nutrient uptake. Irrigation frequency and total water application affect root dispersion and total root length^12^. This determines the physiological processes that are critical to plant growth. To attain higher yields, weed competition during the early phases of crop growth should be minimized^13^. Reduced crop-weed competition resulted in an increase in maize dry matter buildup, which led to improved yield characteristics and grain yield^14^.

The performance of tembotrione as a herbicide has been presented at several conferences and through various scientific papers^15,16^. Topramezone and tembotrione are the new selective, postemergence herbicides introduced for use in maize that inhibit Hydroxy-phenyl pyruvate dioxygenase (4-HPPD) enzyme and the biosynthesis of plastoquinone^15,17^. Tank mixing of these herbicides with lower dose of atrazine was reported to be more effective than application of individual chemical. System Intensification using more biological inputs through best management practices, is the best alternative methodology for sustainable food, nutrition, ecological and health security^18^. The weed menace causes globally 11.5% and at national 10.9% production loss^19^.

Turcicum leaf blight of maize (TLB) is a major foliar disease in maize and caused by *Exserohilum turcicum* (Pass.) Leonard and Suggs. (Synonyms: *Drechslera turcica* (Pass.) Subramanian and Jain; *Bipolar isturcica* (Pass.) Shoemaker; *Helminthosporium turcicum* (Pass.) Leonard and Suggs. The disease is known to affect maize from seedling to harvest. Loss in grain yield will be more if disease occurs before flowering, silking, and grain filling stages due to decreased photosynthetic area. The grain yield loss up to 46.7% has been reported due to TLB in maize^20^. As many as 141 insect pests cause different degrees of damage to maize crops from sowing to harvesting^21^. The fall armyworm, *Spodoptera frugiperda* (E. F. Smith) (Noctuidae: Lepidoptera) has become a serious pest on maize in India and elsewhere. The pest has been very recently reported on maize from Karnataka for the first time in India^22,23^. It is an insect native to tropical and subtropical regions of the Americas. During 2016, the FAW was noticed first in Karnataka and Central maize growing states of India and made the farmers feel panic about the incidence^22^. The fall armyworm larvae are a cosmopolitan, polyphagous pest which can feed on about 80 different plant species including crops such as corn, rice, small millets, sugarcane, alfalfa, soybean, sorghum, cotton, and vegetable crops^23^. The rapid spread of this pest in Indian states and Asian countries is due to its efficient ability to travel and migrate long distances in short time. Pest-related crop losses could amount to enough food to feed more than 1 billion people^24^. It is now obvious that other ways must be used to limit insect damage while avoiding the expense and unfavorable effects associated with synthetic pesticides because the use of synthetic pesticides poses additional obstacles. Integrated Pest Management (IPM) is a combination of several pest management approaches to supplement, minimize, or substitute the reliance of chemical pesticides. IPM encompasses simultaneous management and convergence of strategies, as well as consistent pest and natural enemy surveillance. IPM, on the other hand, is a lot more than merely a resource-saving technology. Due to land degradation, biodiversity loss, and climate change, soil has become one of the most vulnerable resources in the world. Achieving sustainability and increasing agricultural output are both possible with sustainable agricultural techniques. Climate smart agriculture, conservation agriculture and integrated soil fertility management and integrated pest management are agricultural technologies that are frequently marketed as supporting pathways to sustainable intensification (SI). These technologies also include carbon sequestrations, energy budgeting and green economy.

In this context, a field experiment was initiated to enhance sustainable intensification of maize production in rainfed tropics by comparing farmers' methods to precision-conservation best practices comprising green technologies such as crop residue mulching, minimum tillage, integrated soil fertility, weed and pest management options.

## Results

### Grain yield of maize and chickpea

Results of three years pooled data revealed that ecological intensification (EI) treatment which comprises of best tillage, crop residue cover, planting density and genotype, precision nutrient management, application of water at critical growth stages, integrated weed, disease and insect management recorded significantly (p = 0.05) higher grain yield (7560 kg/ha) and stover yield (8757 kg/ha) compared to farmers’ practice (FP) all and other treatments which were deficit in one or another crop management practices (T_3_, T_4_, T_5_, T_6_ and T_8_) (Table 1). In all individual years EI practice recorded significantly higher grain and stover yield. Whereas EI-INM recorded significantly lower grain and stover yield followed by EI-IWM. Similarly test weight and dry matter accumulation at harvest were also significantly higher in EI practice compared to other practices (Table 2).

**Table 1:** Grain and stover yield of maize as influenced by ecological intensification in maize-chickpea system.

Treatments	Grain yield (kg/ha)				Stover yield (kg/ha)			
	2017	2018	2019	Mean	2017	2018	2019	Mean
T_1_ = Farmers’ practice (FP)	7140.8^b^	6601.4^bc^	5427.4^bc^	6389.9^b^	8696.7^b^	7544.6^bc^	7213.6^b^	7818.4^b^
T_2_ = Ecological intensification (EI)	8218.5^a^	7591.8^a^	6869^a^	7559.8^a^	9596.7^a^	8383.7^a^	8290.8^a^	8757.2^a^
T_3_ = EI-RTM	6911.8^b^	6493.6^bc^	6246.7^ab^	6550.7^b^	8400^b^	7306.7^bc^	7520.2^ab^	7742.3^b^
T_4_ = EI-INM	6893^b^	6513.8^bc^	5568.1^bc^	6414.1^b^	4600.1^e^	7388.3^bc^	6784.5^b^	7468.6^bcd^
T_5_ = EI-RPM	6753.5^b^	7010.7^ab^	6238.1^ab^	6667.4^b^	7800^bc^	7939.2^ab^	7303.8^ab^	7681^bc^
T_6_ = EI-RIM	4400.4^d^	6371.2^bc^	5916.2^bc^	5562.6^c^	6493.3^d^	7310^bc^	7281.7^ab^	7028.2^cd^
T_7_ = EI-IWM	4786.9^d^	5537^d^	5328.8^c^	5217.5^c^	6750^d^	6840.3^c^	6952.8^b^	6847.7^d^
T_8_ = EI-IPM	5950.2^c^	6031.2^cd^	5440^bc^	5807.1^c^	7330.5^cd^	7307^bc^	7017^b^	7218^bcd^
Mean of year	6381.7	6552.3	5879.2	6271.2	7912.5	7502.4	7295.5	7570.2
LSD (p = 0.05)	798.2	742.3	756.9	571.6	862.9	752.6	871	634.7

The superscipt letters a, b, c, d indicate significant differences between treatments at p < 0.05.

**Table 2:** Test weight and dry weight of weeds as influenced by ecological intensification in maize-chickpea system.

Treatments	100-seed weight (g)				Total dry matter at harvest (g/plant)			
	2017	2018	2019	Mean	2017	2018	2019	Mean
T_1_ = Farmers’ practice (FP)	30.3^bc^	31.2	29.3^bc^	30.3	271.5^ab^	269.5^b-d^	252.5^ab^	264.5^ab^
T_2_ = Ecological intensification (EI)	31.8^a^	35.2	35.7^a^	34.2	289.5^a^	297.3^a^	268.5a	285.1^a^
T_3_ = EI-RTM	30.5^ab^	30.8	30.9^bc^	30.7	256.2^bc^	277.2^a-c^	261.2^ab^	264.9^ab^
T_4_ = EI-INM	27.6^d^	33.3	30.9^bc^	30.6	212.5^d^	273.5^bc^	254.3^ab^	246.8^b^
T_5_ = EI-RPM	30.5^ab^	32.6	31.8^bc^	31.6	258.5^bc^	288.5^ab^	263.4^ab^	270.1^ab^
T_6_ = EI-RIM	28.2^c^	32.6	32.0^bc^	30.9	242.5^c^	259.2^cd^	259.8^ab^	253.8^b^
T_7_ = EI-IWM	29.4^b^	32.0	30.4^bc^	30.6	249.3^d^	248.5^d^	249.5^ab^	249.1^b^
T_8_ = EI-IPM	29.9	31.7	29.9	30.5	253.4^cd^	257.3^cd^	254.6^ab^	255.1^b^
LSD (p = 0.05)	1.4	NS	3.5	2.1	22.31	21.93	19.56	24.3

The superscipt letters a, b, c, d indicate significant differences between treatments at p < 0.05.

Similarly in the succeeding winter season, significantly (p = 0.05) higher chickpea yield (797 kg/ha) was recorded in EI all the years and the lowest was recorded in EI minus INM (539 kg/ha) (Table 3). Further EI practice showed a greater number of secondary branches and pods per plant compared to remaining practices at p = 0.05 level of significance. In cropping systems, more than one species is involved, and it becomes very difficult to compare the economic produce of different nature. To express the yield advantage, the yields of individual crops in a system were converted into equivalent yield based on their economic value could be expressed in maize equivalent yield (MEY) (Table 3). Higher MEY in a system could be credited to yield advantages attained. Significantly more MEY was obtained in EI practice in both individual years (11,016, 1186, 8852 kg/ha during first, second and third year respectively) and pooled over three years (10,418 kg/ha). Whereas EI-INM recorded the lowest MEY, and it was on par with FP, EI-IWM and EI-IPM during individual years and pooled over three years at p = 0.05 level of significance.

**Table 3:** Chickpea yield and maize equivalent yield as influenced by ecological intensification in maize-chickpea system.

Treatments	Chickpea yield (kg/ha)				Maize equivalent yield (kg/ha)			
	2017	2018	2019	Mean	2017	2018	2019	Mean
T_1_ = Farmers’ practice (FP)	657.2^d^	925.0^bc^	622.5^bc^	734.9^c^	9415.9	9376.4	6983.7	8592.0
T_2_ = Ecological intensification (EI)	808.3^a^	1265.0^a^	793.5^a^	955.6^a^	11,016.0	11,386.8	8852.8	10,418.5
T_3_ = EI-RTM	755.0^bc^	960.0^b^	680.3^bc^	798.4^b^	9525.5	9373.6	7947.5	8948.9
T_4_ = EI-INM	789.4^ab^	895.0^bc^	645.1^bc^	776.5^bc^	4648.5	9198.8	7180.9	7009.4
T_5_ = EI-RPM	795.1^ab^	952.0^bc^	650.5^bc^	799.2^b^	9505.3	9866.7	7864.3	9078.8
T_6_ = EI-RIM	716.1^c^	923.0^bc^	701.2^b^	780.1^bc^	6878.8	9140.2	7669.2	7896.1
T_7_ = EI-IWM	769.1^ab^	912.0^bc^	688.3^bc^	789.8^bc^	7449.3	8273.0	7049.5	7590.6
T_8_ = EI-IPM	720.2^c^	833.0^c^	622.5^bc^	725.2^d^	8443.0	8530.2	6996.3	7989.8
LSD (p = 0.05)	45.7	58.6	79.4	61.233	–	–	–	–

The superscipt letters a, b, c, d indicate significant differences between treatments at p < 0.05.

### Major disease and pest incidence

Fall army worm (FAW-*Spodoptera frugiperda,* J. E. Smith) incidence was significantly higher on maize in EI-RPM (2.7%) followed by FP (1.4%) which was on par with other practices (Table 4). The FAW could threaten the food security and livelihoods of millions of small-scale farmers in Asia as the invasive crop-eating pest is highly likely to spread further from India, with Southeast Asia and South China most at risk. Among the system, FAW least infestation was noticed under EI practice (0.9%). Among the economically important diseases of maize, turcicum leaf blight (TLB: *Setosphaeria turcica* L.), regularly causes varying degrees of yield losses at national level. All the years EI practice recorded significantly lower disease severity score (6.0, 3.7, 4.9 during first, second, and third year respectively) and EI-IPM recorded the highest (8.0, 6.3, 7.2 during first, second and third year respectively) and it was on par with FP. EI showed significantly lower disease severity of 55.00 and 31.67% during the year 2018 and 2019 respectively and pooled mean value 43.33% which was significantly lower over the other treatments. Highest disease severity 65.00 and 72.00% was recorded in farmer’s practices during 2018 and 2019 respectively with mean pooled value 68.50%.

**Table 4:** Turcicum leaf blight and fall armyworm incidence as influenced by ecological intensification in maize-chickpea system.

Treatments	Turcicum leaf blight scores			Fall armyworm scores		
	2018	2019	Mean	2018	2019	Mean
T_1_ = Farmers’ practice (FP)	7.0^b^	7.7^a^	7.4^a^	1.0^bc^	1.7^b^	1.4^bc^
T_2_ = Ecological intensification (EI)	6.0^cd^	3.7^d^	4.9^d^	1.0^bc^	0.7^cd^	0.9^c^
T_3_ = EI-RTM	7.0^b^	7.0^ab^	7.0^ab^	1.3^b^	1.3^bc^	1.3^bc^
T_4_ = EI-INM	7.6^ab^	6.3^bc^	7.0^ab^	1.3^b^	1.3^bc^	1.3^bc^
T_5_ = EI-RPM	7.0^b^	6.0^c^	6.5^bc^	1.2^bc^	1.3^bc^	1.3^bc^
T_6_ = EI-RIM	6.3^c^	6.7^bc^	6.5^bc^	1.3^b^	1.7^b^	1.5^b^
T_7_ = EI-IWM	6.0^cd^	6.3^bc^	6.2^bc^	1.3^b^	1.0^c^	1.2^bc^
T_8_ = EI-IPM	8.0^a^	6.3^bc^	7.2^ab^	2.4^a^	3.0^a^	2.7^a^
LSD (p = 0.05)	0.5	0.9	0.7	0.3	0.6	0.45

The superscipt letters a, b, c, d indicate significant differences between treatments at p < 0.05.

### Weed dynamics

Major weed flora associated with experimental site were grasses, sedges, and broad-leaved weeds. The important grassy weeds observed were *Brachiaria eruciformis* (Trin.) Griseb., *Cynodon dactylon* (L.) Pers*., Dinebra retroflexa* Jacq. and *Cyperus rotundus* L., was under sedges category*.* Among broad leaved weeds, *Ageratum conyzoides* L., *Alternanthera sessilis* H. B and K., *Commelina benghalensis* L., *Euphorbia geniculate* L., *Mollugo disticha* Lamk., *Parthenium hysterophorus* L., *Phyllanthus niruri* Webster and *Corchorus trilocularis* L. were the dominant weeds. All these were smothered effectively in EI practice and their population and dry weight were significantly higher in FP at p = 0.05 level of significance (Table 5).

**Table 5:** Population and dry weight of weeds at 30 days after sowing as influenced by ecological intensification in maize-chickpea system.

Treatments	Total weed population/m^2^				Total weed dry weight (g/m^2^)			
	2017	2018	2019	Mean	2017	2018	2019	Mean
T_1_ = Farmers’ practice (FP)	30.8^b^	25.1^b-d^	35.9^a^	30.6^b^	5.2^b^	4.9^b^	6.1^a^	5.4^b^
T_2_ = Ecological intensification (EI)	4.3^f^	9.7^e^	15.1^c^	9.7^d^	1.0^e^	0.9^e^	1.1^d^	1.0^e^
T_3_ = EI-RTM	20.0^c^	30.5^b^	28.0^cd^	26.2^bc^	4.2^bc^	4.2^bc^	4.5^a-c^	4.3^c^
T_4_ = EI-INM	19.3^cd^	21.2^d^	30.5^bc^	23.7^bc^	3.9^b-d^	2.8^d^	4.0^bc^	3.6^cd^
T_5_ = EI-RPM	15.5^de^	27.3^bc^	29.0^bc^	23.9^bc^	3.4^cd^	3.1^cd^	4.7^a-c^	3.7^cd^
T_6_ = EI-RIM	17.8^cd^	26.4^b-d^	24.1^d^	22.8^c^	3.8^b-d^	3.6^cd^	3.6^c^	3.7^cd^
T_7_ = EI-IWM	47.8^a^	52.6^a^	33.7^ab^	44.7^a^	8.0^a^	8.0^a^	5.4^ab^	7.1^a^
T_8_ = EI-IPM	16.0^cd^	22.6^cd^	23.2^d^	20.6^c^	2.5^d^	2.7^d^	3.4^c^	2.9^d^
LSD (p = 0.05)	4.22	5.12	4.59	6.43	1.31	1.23	1.64	0.71

The superscipt letters a, b, c, d indicate significant differences between treatments at p < 0.05.

### Economic analysis of practices

The net return was calculated treatment wise by subtracting the total cost of cultivation from gross returns and expressed in rupees per hectare (Rs./ha). Whereas benefit–cost ratio was worked out by dividing gross returns from cost of cultivation. To assess the advantage of any production system, it is finally the economics of the system which plays a major role in its acceptance by the farmers. Net returns obtained in different cultivation practices varied significantly. Each individual year and pooled data showed that EI practice recorded significantly more net return (Rs. 76,338, 83,469 and 82,011 during first, second and third year respectively), and benefit–cost ratio (1.8, 3.2 and 2.7 during first, second and third year respectively) (Table 6). The lowest net returns were obtained in FP EI-integrated weed management (Rs.51354.7/ha) followed by EI-recommended irrigation management (Rs. 56,015.3/ha), integrated pest management (Rs. 59,569.7/ha) and farmers’ practice (Rs. 67,357.7/ha) over three years. Pooled net returns over three years also followed a similar trend with the highest net returns of Rs.80606/ha in EI practice. Further benefit–cost ratio of different cultivation practices varied significantly. In the first year and pooled data showed that B-C ratio was significantly higher FP which was on par with EI-INM and EI-RPM. The EI-IWM recorded the lowest B-C ratio followed by EI practice. B-C ratio varied year to year among the practices depending on yield obtained and cost invested at p = 0.05 level of significance.

**Table 6:** Economics of maize as influenced by ecological intensification in maize-chickpea system.

Treatments	Net returns (Rs./ ha)				B-C ratio			
	2017	2018	2019	Mean	2017	2018	2019	Mean
T_1_ = Farmers’ practice (FP)	67829^ab^	73623^b-d^	60621^bc^	67,357.7^bc^	5.2^a^	3.3^ab^	2.4	3.6^a^
T_2_ = Ecological Intensification (EI)	76338^a^	83469^a^	82011^a^	80,606.0^a^	1.8^cd^	3.2^a-c^	2.7	2.6^d^
T_3_ = EI-RTM	64081^b^	68898^c-e^	73387^a-c^	68,788.7^bc^	3.2^bc^	3.0^b-d^	2.6	2.9c
T_4_ = EI-INM	68497^ab^	76220^a-c^	63293^bc^	69,336.7^bc^	3.9^b^	3.3^ab^	2.5	3.2^b^
T_5_ = EI-RPM	62793^b^	80172^ab^	76523^ab^	73,162.7^ab^	3.4^bc^	3.5^a^	2.8	3.2b
T_6_ = EI-RIM	33700^d^	66939^de^	67407^bc^	56,015.3^cd^	3.8^bc^	2.9^cd^	2.5	3.1b^c^
T_7_ = EI-IWM	39727^d^	56591^f^	57746^c^	51,354.7^cd^	1.9^cd^	2.8^d^	2.3	2.3e
T_8_ = EI-IPM	54350^c^	63499^ef^	60860^bc^	59,569.7^c^	2.5^c^	2.9^cd^	2.4	2.6^d^
LSD (p = 0.05)	8393	7877	14,373	8,550.5	1.3	0.35	NS	0.26

The superscipt letters a, b, c, d indicate significant differences between treatments at p < 0.05.

### Energetic analysis of practices

Input energy which considers labor engaged organics, fuel, seeds, fertilizers pesticides and irrigation cost varied in different practices. Significantly lower input energy use was in FP (17,855.2 MJ/ha), whereas significantly higher energy input was in EI (24,647 MJ/ha)which was on par with remaining practices (Tables 7 and 8). Whereas total output energy produced was the highest in EI (220,590 MJ/ha) and lower output energy was recorded in EI–INM (149,255 MJ/ha). Input and output energy calculated for remaining practices were on par with each other. Net energy also followed a similar trend to that of output energy. EI recorded higher net energy and on the contrary EI–INM recorded lower net energy. Energy use efficiency is cultural energy utilized through inputs and energy produced as products. FP revealed higher energy use efficiency followed by EI. Whereas lower energy use efficiency was reported in EI–INM. Similarly higher energy productivity was also recorded in FP followed by EI. Specific energy is energy required to produce per kilo of main product. So, it is understood that the lower the specific energy, the better the practice. Lower specific energy was recorded in FP and was followed by EI. Whereas higher specific energy was noticed is EI–INM. Efficient use of energy helps to achieve increased production and productivity and contributes to the economy, profitability, and competitiveness of agriculture sustainability in rural areas^25,26^.

**Table 7:** Contribution of different production factors towards total input energy (MJ/ha).

Treatments	Labor	Mach	Diesel	FYM	Seeds	Fertilizers	Micronutrients	Weedicides	Insecticides	Fungi	Irrigation	Total input
T_1_ = Farmer’s practice (FP)	1565.9	1522.0	2252.4	757.8	304.0	10,913.1	0.0	0.0	540.0	0.0	0.0	17,855.2
T_2_ = Ecological Intensification (EI)	1318.5	1814.4	2534.0	2273.3	342.0	9921.0	627.0	540.0	432.0	540.0	4305.0	24,647.1
T_3_ = EI-RTM	1196.8	1522.0	2534.0	2273.3	342.0	9921.0	627.0	540.0	1080.0	540.0	4305.0	24,881.0
T_4_ = EI-INM	1172.3	1814.4	2534.0	757.8	342.0	10,913.1	627.0	540.0	1080.0	540.0	4305.0	24,625.5
T_5_ = EI-RPM	1261.0	1814.4	2252.4	2273.3	304.0	9921.0	627.0	540.0	1080.0	540.0	4305.0	24,918.1
T_6_ = EI-RIM	1271.7	1814.4	2534.0	2273.3	342.0	9921.0	627.0	540.0	1080.0	540.0	0.0	20,943.3
T_7_ = EI-IWM	1176.7	1814.4	2534.0	2273.3	342.0	9921.0	627.0	0.0	1080.0	540.0	4305.0	24,613.3
T_8_ = EI-IPM	1167.3	1814.4	2534.0	2273.3	342.0	9921.0	627.0	540.0	540.0	0.0	4305.0	24,063.9

**Table 8:** Energetic of maize as influenced by ecological intensification in maize-chickpea system.

Treatments	Input energy (MJ/ha)	Output energy (MJ/ha)	Net energy (MJ/ha)	Energy use efficiency	Energy productivity (kg MJ^−1^)	Specific energy (MJ kg^−1^)
T_1_ = Farmers’ practice (FP)	17,855.2	191,660.3	173,805.1	10.73	0.36	2.79
T_2_ = Ecological intensification (EI)	24,647.1	220,589.9	195,942.8	8.95	0.31	3.26
T_3_ = EI-	24,881.0	193,075.5	168,194.5	7.76	0.26	3.80
T_4_ = EI-INM	24,625.5	149,254.8	124,629.3	6.06	0.20	5.10
T_5_ = EI-RPM	24,918.1	194,021.8	169,103.7	7.79	0.27	3.74
T_6_ = EI-RIM	20,943.3	169,623.8	148,680.5	8.10	0.27	3.77
T_7_ = EI-IWM	24,613.3	166,705.0	142,091.7	6.77	0.21	4.72
T_8_ = EI-IPM	24,063.9	175,589.4	151,525.5	7.30	0.24	4.14

### Soil fertility status

**Figure 1:**
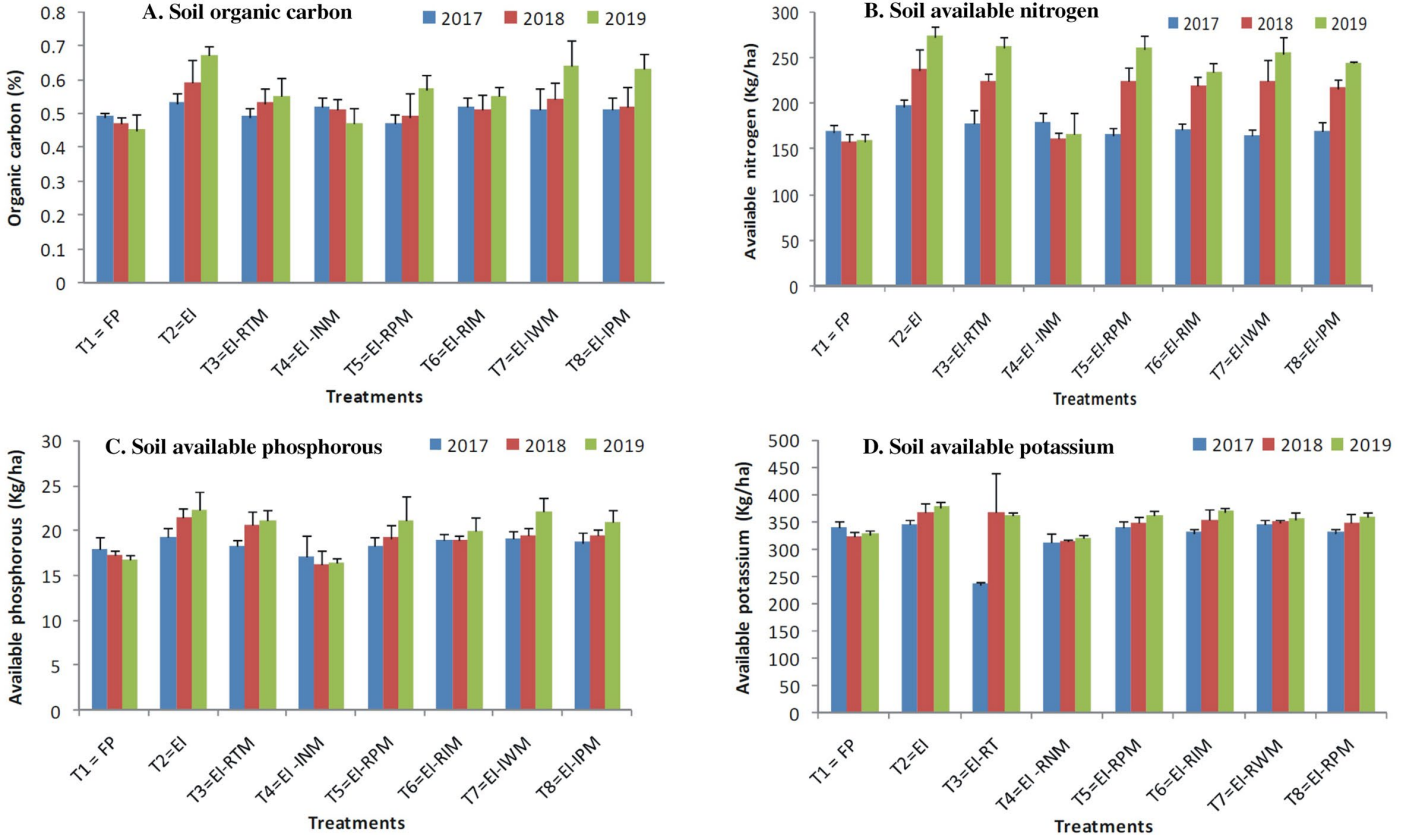
Soil organic carbon sequestration and available nutrients contents after the harvest of the experiments during the experimental period. (**A**) Soil organic, (**B**) soil available nitrogen, (**C**) soil available phosphorous and (**D**) soil available potassium.

**Figure 2:**
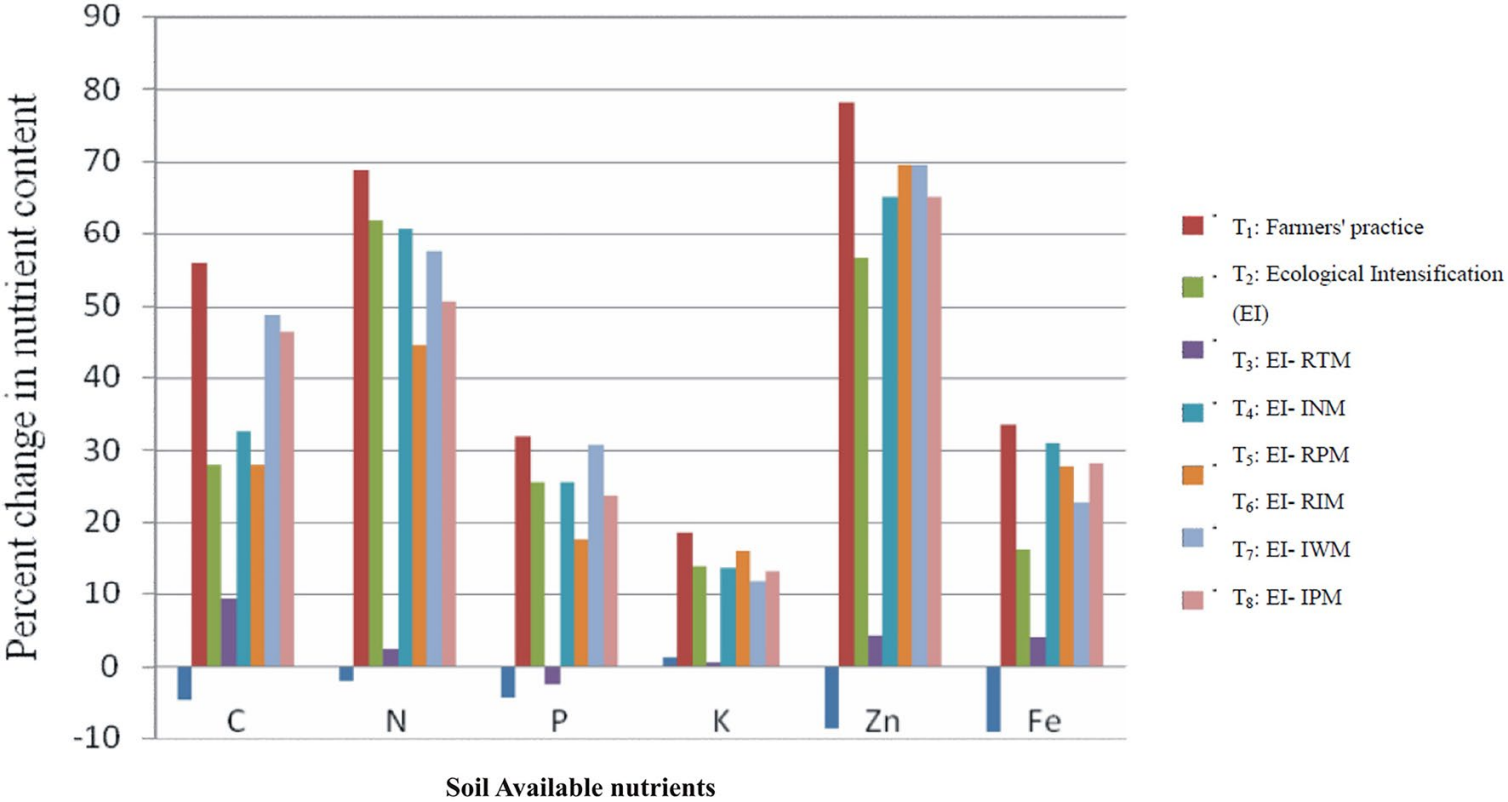
Percent change in soil organic carbon, available nitrogen, phosphorous, potassium, zinc, and iron over the period of experimentation.

At initial stage of experimentation and after completion of each cycle of maize–chickpea cropping system, soil samples were analyzed for nutrient status in soil, the results showed that soil organic carbon (SOC) content in soil at the beginning of experimentation was deficit (0.43%) and it reached to medium (0.49 to 0.52%) in all the practices wherever crop residue was retained (Table 9). There was further improvement in soil organic carbon over the years and significantly more so in EI practice. Similar trend was recorded with respect to improvement soil available nutrient content in soil. EI practice recorded significant amount of organic carbon, available nitrogen, phosphorus, potassium, zinc, and iron after completion of third cycle of experimentation (0.60%, 235.3 kg/ha,21.0 kg/ha,363.2 kg/ha,0.52 ppm and 5.2 ppm, respectively) which account to 27,46,23,10,68 and 22% improvement respectively over FP (Tables 9 and 10, Figs. 1 and 2). The comparison was made with initial value and results showed that FP recorded negative for organic carbon, available nitrogen, and phosphorus, DTPA extractable zinc and iron (Fig. 2). Whereas EI practice showed significant improvement of nutrient build up in soil. Further initial value in EI-INM did not show any improvement in nutrient status of soil compared to FP. And it recorded lower phosphorus and potash content in soil compared to FP. Whereas, other practices recorded increased nutrient status over the FP, but rate of increased linearity was less compared to EI practice. In FP, SOC, available nitrogen, and phosphorus remained deficit all through the years and it was on par with EI-INM practice.

**Table 9:** Organic carbon and available nitrogen in soil as influenced by different ecological intensification treatments.

Treatments	Organic carbon (%)				Available Nitrogen (kg/ha)			
	2017	2018	2019	Mean	2017	2018	2019	Mean
T_1_ = Farmers’ practice (FP)	0.46	0.47^a^	0.45^b^	0.47^b^	168.5^bc^	157.3^b^	158.5^d^	161.4^b^
T_2_ = Ecological intensification (EI)	0.51	0.59^a^	0.67^a^	0.60^a^	196.7^a^	236.5^a^	272.7^a^	235.3^a^
T_3_ = EI-RTM	0.49	0.53^ab^	0.55^ab^	0.52^ab^	177.5^ab^	223.6^ab^	261.3^ab^	220.8^ab^
T_4_ = EI-INM	0.52	0.51^ab^	0.47^b^	0.50^ab^	179.5^ab^	161.5^b^	165.6^d^	168.9^b^
T_5_ = EI-RPM	0.49	0.52^ab^	0.57^ab^	0.51^ab^	165.2^bc^	223.6^ab^	259.6^a-c^	216.1^ab^
T_6_ = EI-RIM	0.52	0.51^ab^	0.55^ab^	0.53^ab^	171.1 ^bc^	219.5^ab^	233.6^c^	208.1^ab^
T_7_ = EI-IWM	0.51	0.54^ab^	0.64^ab^	0.56^ab^	164.5 ^bc^	224.2^ab^	254.5^a-c^	214.4^ab^
T_8_ = EI-IPM	0.51	0.52^ab^	0.63^ab^	0.55^ab^	169.3 ^bc^	216.5^ab^	243.4^bc^	209.7^ab^
LSD (p = 0.05)	NS	0.11	0.13	0.12	22.61	27.11	24.31	24.74

The superscipt letters a, b, c, d indicate significant differences between treatments at p < 0.05.

**Table 10:** Available phosphorus and potassium in soil as influenced by different ecological intensification treatments.

Treatments	Available phosphorus (kg/ha)				Available potassium (kg/ha)			
	2017	2018	2019	Mean	2017	2018	2019	Mean
T_1_ = Farmers’ practice (FP)	17.9^ab^	17.2^cd^	16.8^b^	17.3 ^bc^	339.5^ab^	322.5 ^bc^	326.5^b^	329.5^bc^
T_2_ = Ecological intensification (EI)	19.3a	21.5a	22.3^a^	21.0^a^	345.5^a^	367.5^a^	376.5^a^	363.2^a^
T_3_ = EI-RTM	18.3^ab^	20.6^ab^	21.2^ab^	20.0^ab^	234.9^c^	366.3^ab^	361.8^ab^	321.0^bc^
T_4_ = EI-INM	17.1^b^	16.3^d^	16.5^b^	16.6^c^	311.2^b^	315.0^c^	319.1^bc^	315.1^c^
T_5_ = EI-RPM	18.2^ab^	19.2^a-c^	21.2^ab^	19.5^ab^	338.5^ab^	348.5^ab^	361.3^ab^	349.4^ab^
T_6_ = EI-RIM	18.9^ab^	18.9^bc^	19.9^ab^	19.2^ab^	329.6^ab^	352.6^ab^	368.5^ab^	350.2^ab^
T_7_ = EI-IWM	19.1^ab^	19.5^a-c^	22.1^ab^	20.2^ab^	344.5^ab^	349.5^ab^	354.9^ab^	349.6^ab^
T_8_ = EI-IPM	18.7^ab^	19.5^a-c^	20.9^ab^	19.7^ab^	329.6^ab^	348.5^ab^	359.6^ab^	345.9^ab^
LSD (p = 0.05)	1.78	2.31	2.45	2.33	27.5	28.35	24.66	27.24

The superscipt letters a, b, c, d indicate significant differences between treatments at p < 0.05.

### Enzymatic activity in soil

Soil dehydrogenase and phosphatase enzymatic activity was improved in EI practice over the years and improvement in each year was significant compared to FP (Table 12). In EI practice, pooled data showed that dehydrogenase and phosphatase increase was 26.6 and 33.7% over FP. EI-INM practice found on par with FP with respect to these two enzymatic activities in soil in all the years.

## Discussion

### Sustainability of productivity of maize–chickpea system

Grain yield of maize was significantly higher in EI practice as it comprised all recommended agronomic practices which included conservation tillage, crop residue cover, optimum planting density, precision nutrient management, application of water at critical growth stages, integrated weed, disease, and insect compared to FP and other treatments which were deficit in one or another best crop management practices. Grain yield was significantly higher in residue cover in comparison with conventional tillage and residue removed^27^. But in the case of EI-IWM, grain yield was not significantly influenced by residue cover (Table 1). In all three individual years also, EI practice recorded higher grain and stover yield due to improved test weight of grain and decreased weed population and their dry weight. The findings of higher maize yield under EI are in close agreement with other findings of reports of higher maize yield in conservation practice^28–31^. The higher yield of maize under EI could be attributed to the compound effect of better establishment of the crop due to favorable soil temperature and moisture conditions in soil, additional nutrients^32^, reduced competition for resources and improved bio-physico-chemical soil health over conventional farmers practice^3,33^. Precision-conservation agriculture, scale-appropriate mechanization and integrated nutrient management can help support sustainable intensification of maize-based cropping systems, helping to improve efficient use of soil, labor, water and nutrients^34^. Like maize yield, significantly higher chickpea yield (797 kg ha^−1^) was also recorded in EI all the years (Table 3). Further it showed a greater number of secondary branches and pods per plant compared to remaining practices as result of better soil fertility and soil moisture retention. Surface maintained crop residues act as mulch and therefore reduce soil water losses through evaporation and maintain a moderate soil temperature regime^35^. In cropping systems, more than one species is involved, and it becomes very difficult to compare the economic produce of different nature. To express the yield advantage, the yields of individual crops in a system were converted into equivalent yield based on their economic value could be expressed in maize equivalent yield (MEY). Higher MEY in a system could be credited to yield advantages attained. Significantly more MEY was obtained in was also significantly higher in EI practice in all individual years (11,016, 1186, 8852 kg ha^−1^ during first, second and third year respectively) and pooled over three years (10,418 kg ha^−1^) (Table 3). Whereas EI-INM recorded the lowest maize equivalent yield, and it was on par with FP, EI-IWM and EI-IPM during individual years and pooled over three years due to poor performance of maize–chickpea cropping systems in these practices. INM practice obtained significantly p ≤ 0.01) higher grain yield (8.42 tons ha^−1^) than farmer dose of fertilizer^27^.

Crop residue retention had lot of advantages as part of improvement in soil organic carbon, available nutrients and soil microbial activity are concerned. EI followed some principle of conservation agriculture (CA) which has three principles: (1) establishing crops with reduced tillage, (2) residue mulches on the soil surface, and (3) crop rotations^36^. The crop residues become mulch over the soil surface that protects the soil productive layer against runoff reducing the nutrient loss and erosion through runoff^37,38^ and increases the percentage of organic matter in the soil layer^39^. In EI practice, INM, IWM and IPM were the major tools for best crop production. They embrace soil, nutrient, water, crop, and vegetation management practices, tailored to a particular cropping and farming system, undertaken with the aim of improving and sustaining soil fertility and land productivity, and reducing environmental degradation. Precision INM aims to optimize the condition of the soil, about its physical, chemical, biological, and hydrological properties, for the purpose of enhancing farm productivity, whilst minimizing land degradation. Whereas EI-INM recorded lower grain and stover yield followed by EI-IWM as they recorded lower test weight and dry matter accumulation at harvest. It is well known that each of the nutrient elements plays a major role in growth and development of the plants, and when present in deficient quantities can reduce growth and yields^40^. One of the primary goals of the integrated strategy is to minimize the use of exogenous agriculture inputs like synthetic fertilizers and pesticides^41^. INM relies largely on the balanced application of appropriate fertilizers. Excess fertilizer usage does not result in significantly higher crop absorption of nutrients or yields. Furthermore, excessive nutrient applications are monetarily unproductive and may adversely affect the ecosystem. On the other hand, inadequate application might affect the crop growth and reduce yields in the short term while jeopardizing sustainability through soil exploitation, over time. Secondary nutrients and micronutrients that are also easily obtainable from organic fertilizers should also be included in a balanced fertilization^42^. The use of exclusively inorganic fertilizers causes nutritional imbalance, resulting in poorer yields and reduced yearly returns^43^.

### Weed management

Weed management in maize can be highly critical due to wider row spacing. Manual control of weeds growing between the rows is labor intensive. Herbicides weed control is an important alternative to manual weeding because it is cheaper, faster and gives better weed control in maize^44^. Weed problems are more severe during continuous rains in early stages of maize growth which cannot be controlled by traditional and cultural practices alone due to too much wetness and difficulty in hand weeding. Weeds reduce maize yield from 33 to 50% depending upon weed species and density^45^. Weeds compete with crops for light, moisture, space, and plant nutrients and consequently interfere with the normal growth of crops. It is known that there is a critical crop-weed competition period with grain losses reaching between 28 and 100% if weeds are not controlled^46,47^. Control of weeds in maize is, therefore, very important for obtaining higher productivity. Much of previous crop residue covered the soil and thereby reduced the weed menace in all the treatments except FP where residue was not retained on soil. Apart from FP and EI-IWM practices, which were affected by crop residue cover, the maize crop cultivated under the EI practice had a decreased population of weeds and their dry weight (Table 5). Establishment of weeds and emerged weeds were controlled by application of tembotrione at 2 to 3 leaf stage of weeds resulting reduced dry weight of weeds. Weed control practices in maize resulted in 77 to 96.7% higher grain yield than the weedy check^48,49^. EI-IWM showed more weed menace as IWM practice was not affected and only cultural method such as hand weeding has been done which was less effective under system of soil cover with residue. Perennial weeds have a persistence effect under EI-IWM and FP. The performance of Tembotrione and Topramezone as effective herbicides has been already established^15,16,50^. However, there are reports of residual toxicity and there is need to use of alternative herbicides to avoid build –up of residue in soil. Topramezone and tembotrione are the new selective, post emergence herbicides introduced for use in maize that inhibit Hydroxy-phenyl pyruvate dioxygenase (4-HPPD) enzyme and the biosynthesis of plastoquinone^15,17^. Residues of tembotrione and TCMBA persisted up to 60 days in soil and tembotrione show maximum leaching up to 25 cm in soil depth^51^. Therefore, tank mixing of these post emergence herbicides with lower dose of atrazine was reported to be more effective than application of individual chemical.

### Management of Fall armyworm

Another two major pests of maize are recent invasive Fall armyworm (FAW) and turcicum leaf blight (TLB) and they influence significantly maize productivity. FAW incidence was significantly higher on maize in EI-IPM (2.7%) practice. In other practices the incidence of FAW was on par with FP (1.4%). IPM is also influenced by several factors. Each grower has their own strategy for producing crops, minimizing losses, and making a profit in a manner that is acceptable to the retailer, safe for the consumers, and less disruptive to the environment. In other words, IPM is an approach to manage pests in an economically viable, socially acceptable, and environmentally safe manner^52,53^. Among the systems, FAW least infestation was noticed under EI practice (0.9%) as control measures were initiated at right time with cultural and chemical measures as part of IPM strategy. Among the economically important diseases of maize, turcicum leaf blight (TLB: *Setosphaeria turcica*), regularly cause varying degrees of yield losses at national level. All the years EI practice recorded significantly lower disease severity score (6.0, 3.7, 4.9 during first, second and third year respectively) as integrated disease management was part of EI practice (Table 4). EI-RPM recorded the highest (8.0, 6.3, 7.2 during first, second and third year respectively) and it was on par with FP. Whereas, under uncontrolled condition yield loss could reach upto 4.5%.

### Soil fertility status

Organic carbon (OC) content in soil at the beginning of experimentation was deficit and it reached to medium (0.49 to 0.52%) in all the practices wherever crop residue was retained. There was further improvement in organic carbon over the years and more so in EI practice due to application of organics and crop residue retention which resulted in significant improvement in organic carbon, available nitrogen, phosphorus, potassium, zinc, and iron after completion of third cycle of experimentation (0.60%, 235.3 kg/ha, 21.0 kg/ha, 363.2 kg/ha, 0.52 ppm and 5.2 ppm respectively). It accounted for 27, 46, 23, 10, 68 and 22% improvement respectively over FP. Introduction of crop residue in the soil offers the best means to restore carbon in agriculture soils^54^. After comparing with initial value, FP recorded depletion of soil organic carbon, available nitrogen, and phosphorus, DTPA extractable zinc and iron (Table 11 and Fig. 2). Whereas EI practice showed significant improvement of nutrient build-up in soil due to crop residue retention and addition of recommended organic source in the form of farmyard manure (FYM-10.0 tons/ha). High yielding crops like maize require large amounts of mineral nutrients from soil which require proper nutrient management strategy that minimize loss and maximize the efficiency of use^27^. Except in FP, other practices were also covered with crop residue and FYM and that is why they are shown improved soil nutrient status compared to FP. Further EI-INM and FP recorded lower phosphorus and potash content in soil. Whereas other practices recorded increased nutrient status over the FP, but the rate of increased linearity was less compared to EI practice. In FP, soil OC, available nitrogen and phosphorus remained deficit all through the years due to negligible application of organic manures and no soil cover with crop residue. Soil dehydrogenase and phosphatase enzymatic activity were improved in EI practice over the years and improvement in each year was significant compared to FP (Table 12). In EI practice pooled data showed that dehydrogenase and phosphatase increase was 26.6 and 33.7% over FP due to linear increase in organic carbon and its mineralization over the years. EI-INM practice found on par with FP with respect these two enzymes activity in soil in all the years. Because of high organic matter and nutrients in EI practice, the population of bacteria, fungi and actinomycetes were increased. And their activity in the form of dehydrogenase, alkaline phosphatase and acid phosphatase are the enzymes responsible for converting organic P into available form^55^. The increased population of microorganisms hasa role in yield maximization through increased nutrient availability^56^. Higher activity of dehydrogenase enzyme and microbes in organic -amended soil than in unamended soil was reported by many researchers^57,58^.

**Table 11:** DTPA extractable zinc and iron (ppm) in soil as influenced by different ecological intensification treatments.

Treatments	DTPA extractable zinc (ppm)				DTPA extractable iron (ppm)			
	2017	2018	2019	Mean	2017	2018	2019	Mean
T_1_ = Farmers’ practice (FP)	0.22^b^	0.21^c^	0.22^e^	0.22^c^	4.23^b^	4.22^b^	4.35^c^	4.27^b^
T_2_ = Ecological intensification (EI)	0.43^a^	0.42^a^	0.41^a^	0.42^a^	4.95^a^	5.31^a^	5.42^a^	5.23^a^
T_3_ = EI-RTM	0.34^ab^	0.34^ab^	0.36^a-e^	0.35^ab^	4.35^ab^	4.51^b^	4.72^bc^	4.53^ab^
T_4_ = EI-INM	0.23^b^	0.24^bc^	0.24^b-e^	0.24^bc^	4.11^b^	4.32^b^	4.22^c^	4.22^b^
T_5_ = EI-RPM	0.38^ab^	0.39^ab^	0.38^a-d^	0.38^ab^	4.62^ab^	4.69^ab^	5.32^a^	4.88^ab^
T_6_ = EI-RIM	0.37^ab^	0.41^ab^	0.39^ab^	0.39^ab^	4.68^ab^	4.78^ab^	5.19^ab^	4.88^ab^
T_7_ = EI-IWM	0.38^ab^	0.41^ab^	0.39^a-c^	0.39^ab^	4.58^ab^	4.74^ab^	4.98^ab^	4.77^ab^
T_8_ = EI-IPM	0.39^ab^	0.38^ab^	0.38^a-d^	0.38^ab^	4.56^ab^	4.83^ab^	5.21^ab^	4.87^ab^
LSD (p = 0.05)	0.094	0.11	0.13	0.11	0.61	0.58	0.51	0.69

The superscipt letters a, b, c, d indicate significant differences between treatments at p < 0.05.

**Table 12:** Dehydrogenase and phosphatase in soil as influenced by different ecological intensification treatments.

Treatments	Dehydrogenase activity (mg TPF/g/day)				Phosphatase (mg P-nitrophenol/g/h g^−1^ h^−1^)			
	2017	2018	2019	Mean	2017	2018	2019	Mean
T_1_ = Farmers’ practice (FP)	26.5^bc^	27.4^c^	27.1^c^	27.00^c^	85.3^c^	81.5^d^	83.6^d^	83.47^d^
T_2_ = Ecological intensification (EI)	32.5^a^	33.6^a^	36.5^a^	34.20^a^	103.5^a^	112.6^a^	116.9^a^	111.00^a^
T_3_ = EI-RTM	29.6^ab^	29.3^a-c^	32.5^b^	30.47^a-c^	92.6^bc^	99.3^bc^	105.6^bc^	99.17^b^
T_4_ = EI-INM	27.6^b^	28.5^bc^	29.2^c^	28.43^bc^	91.3^bc^	86.5^d^	87.9^d^	88.57^cd^
T_5_ = EI-RPM	29.3^ab^	29.6^a-c^	33.1^ab^	30.67^ab^	92.3^bc^	96.3c	98.5c	95.70^bc^
T_6_ = EI-RIM	31.3^ab^	32.6^ab^	34.2^ab^	32.70^ab^	98.3^ab^	99.5^bc^	103.5^bc^	100.43^b^
T_7_ = EI-IWM	30.2^ab^	31.9^a-c^	34.1^ab^	32.07^ab^	95.6^ab^	103.5^bc^	109.3^ab^	102.80^b^
T_8_ = EI-IPM	31.3^ab^	33.1^ab^	33.9^ab^	32.77^ab^	97.3^ab^	106.5^ab^	108.5^ab^	104.10^ab^
LSD (p = 0.05)	3.31	4.25	3.11	4.10	7.61	8.58	8.52	7.69

The superscipt letters a, b, c, d indicate significant differences between treatments at p < 0.05.

### Energetics analysis in different practices

**Figure 3:**
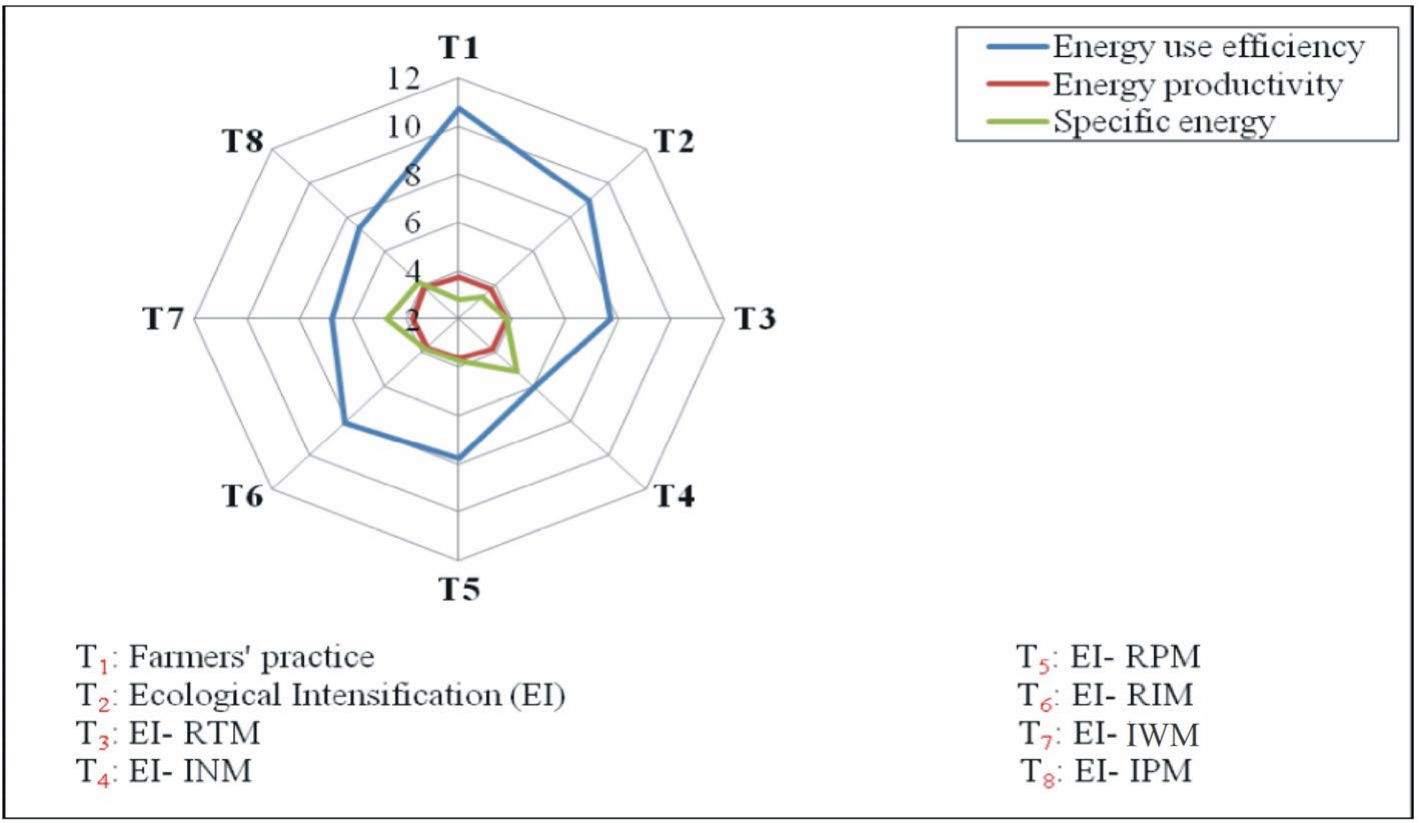
Average energy use efficiency, energy productivity and specific energy transformed in different practices.

Input energy which takes into variable cost incurred on labor engaged, organics, fuel, seeds, fertilizers pesticides and irrigation varied in different practices. Significantly lower input energy use was in farmers’ practice (17,855.2 MJ/ha)as there was no investment in crop residue application and potash fertilizers (Table 8 and Fig. 3). Further pesticide, micronutrient and fertilizer purchase cost was also less. However, there was more investment on land preparation in FP. Land preparation and planting practices are considered the next most important energy component investment in conventionally managed agricultural systems^59,60^. On the contrary, higher energy input was in EI (24,647 MJ/ha) which was on par with remaining practices (Table 8). In Iran, maize production systems total energy input was 39,295.50 MJ/ha^61^. Whereas total output energy produced was the highest in ecological intensification (220,590 MJ/ha) and lower output energy was recorded in EI–NRM (149,255 MJ/ha). Whereas in Iran, total output energy recorded was 58,065 MJ/ha^61^. Input and output energy calculated for remaining practices were on par with each other. It is understood that the output energy depends upon the grain and stover yield of the crop. The higher the grain and stover yield, the higher will be the output energy. The lower output energy in EI–INM was due to low yield as results of deficiency of nutrients particularly potash and zinc. Nutrient management is very important since it utilizes almost 70% of total input energy used in maize production^62^. Similarly net energy also followed a similar trend to output energy i.e,. EI recorded higher net energy. The highest share of energy consumed was recorded for N fertilizer (39%) which is a nonrenewable resource and agrochemicals altogether consumed 46.42% energy which was very much percent of input energy in this agro-ecosystems^61^. Conversely EI–INM recorded lower net energy. Energy use efficiency is cultural energy utilized through inputs and energy produced as products are calculated. The higher energy use efficiency means better utilization of energy. FP revealed higher energy use efficiency followed by ecological intensification. Energy efficiency (output-input ratio) was 1.48 in Iran^61^. Whereas lower energy use efficiency was reported in EI–INM. Similarly higher energy productivity was also recorded in farmers’ practice followed by EI. Specific energy is energy required to produce kg of main product. So, it is understood that the lower the specific energy, the better is the treatment. Lower specific energy was recorded in Farmers’ practice and was followed by ecological intensification treatment. On an average specific energy use was observed as 9.95 MJ/kg^61^. Whereas higher specific energy was noticed is EI–Nutrient management. In conservation agriculture, energy efficiency was higher^63^.

Energy productivity is defined as the quantity of physical output obtained per every unit of input. The higher energy productivity indicates for every unit of input energy there is a higher quantity of physical output. Lower energy productivity was noticed in EI-Nutrient management. Specific energy is energy required to produce kg of main product. So, it is understood that the lower the specific energy, the better is the treatment. Lower specific energy was recorded in Farmers’ practice and was followed by ecological intensification treatment. Whereas higher specific energy was noticed is EI–Nutrient management. It was reported higher input energy, output energy and energy balance in higher nutrient levels and higher energy use efficiency and energy productivity in lower nutrient levels in both maize and wheat crop^64^. Several workers reported higher output energy and net energy return in site specific nutrient management compared to farmer practice and RDF due to higher yield levels in precision nutrient management practices^65,66^. It was also found that higher input energy, output energy and net energy in higher fertility levels compared to lower fertility levels^67^.

### Economic analysis of practices

The net return was calculated treatment wise by subtracting the total cost of cultivation from gross returns and expressed in rupees per hectare (Rs. ha^−1^). Whereas benefit–cost ratio was worked out by dividing gross returns from cost of cultivation. To assess the advantage of any production system, it is finally the economics of the system which plays a major role in its acceptance by the farmers. Net returns obtained in different cultivation practices varied significantly. Lower production costs and energy use, and boost income, while improving system resilience^68–70^. Reduced tillage systems can aid system yields in rice-maize in rotations in Bangladesh^71^. Each individual year and pooled data showed that EI practice recorded significantly more net return (Rs. 76,338, 83,469 and 82,011 during first, second and third year respectively), and benefit–cost ratio (1.8, 3.2 and 2.7 during first, second and third year respectively) (Table 6). Pooled data showed that EI practice recorded higher net returns (Rs. 86,606/ha). The lower net returns were obtained in EI-integrated weed management (Rs. 51,354.7/ha) which was on par with FP, EI-recommended irrigation management and EI-integrated pest management. Further benefit–cost ratio of different cultivation practices varied significantly. In the first year and pooled data showed that the B:C ratio was significantly higher FP which was on par with EI-INM and EI-RDM. EI-IWM recorded the lowest B:C ratio followed by EI practice. B-C ratio varied year to year among the practices depending on yield obtained and cost invested.

Similarly, net return (Rs.76338 ha^−1^) was significantly higher in EI compared to the rest of treatments. The lowest yield (1916 kg ha^−1^), net return (Rs. 18,497 ha^−1^) and B:C ratio was obtained in EI minus INM (T4). Similarly, significantly higher chickpea yield (797 kg ha^−1^) was recorded in EI all the years and the lowest was recorded in EI minus INM (539 kg ha^−1^). Furthermore, EI practice showed a greater number of secondary branches and pods per plant compared to remaining practices. In cropping systems, more than one species is involved, and it becomes very difficult to compare the economic produce of different nature. To express the yield advantage, the yields of individual crops in a system are converted into equivalent yield based on their economic value could be expressed in maize equivalent yield (MEY). Higher MEY in a system could be credited to yield advantages attained. Significantly more MEY was obtained in was also significantly higher in EI practice in both individual years (11,016, 1186, 8852 kg ha^−1^ during first, second and third year respectively) and pooled over three years (10,418 kg ha^−1^). Whereas EI-INM recorded the lowest maize equivalent yield, and it was on par with FP, IWM and IPM during individual years and pooled over three years. Tembotrione herbicidal treatments had only 0.131-0.201 times of application cost than that under manual weeding thrice. Herbicidal treatments resulted in 9.7–13.3 times higher marginal benefit:cost ratio over hand weeding thrice^72^.

## Conclusion

Ecological intensification (EI) practice recorded significantly higher grain yield, maize equivalent yield, net return, net energy from maize–chickpea sequence cropping system compared to farmers’ practice (FP) and other practices which were deficit in one or the other crop management practice. Further, EI practice, recorded significant amount of soil organic carbon, available plant nutrients and microbial activity after completion of third crop sequence indicating linear reduction of carbon footprints by sequestration and improving ecological sustainability.

## Methods

### Experimental materials

A field experiment was carried out at the Main Agricultural Research Station (15° 12′ N, 74° 59′ E), Dharwad, Karnataka, India, for three consecutive years from 2017–2018 to 2019–2020 for sustainable intensification of climate-smart maize–chickpea cropping system in northern transition zone of Karnataka under rain fed condition. The soil at the location of the experiment site was a medium black clay with a pH of 7.6 and electrical conductivity of 0.35 dS m^−1^. The soil had a medium level of organic carbon (0.51%), as well as medium levels of available nitrogen (296 kg ha^−1^), phosphorus (28 kg ha^−1^), and potassium (283 kg ha^−1^). The JG-11 chickpea variety and the University's single cross maize hybrid, GH-150125, were selected as the experimental seed materials. Experimental research and field studies on cultivated varieties complied with relevant institutional, national, and international guidelines and legislation.

### Climatic condition during cropping years

**Figure 4:**
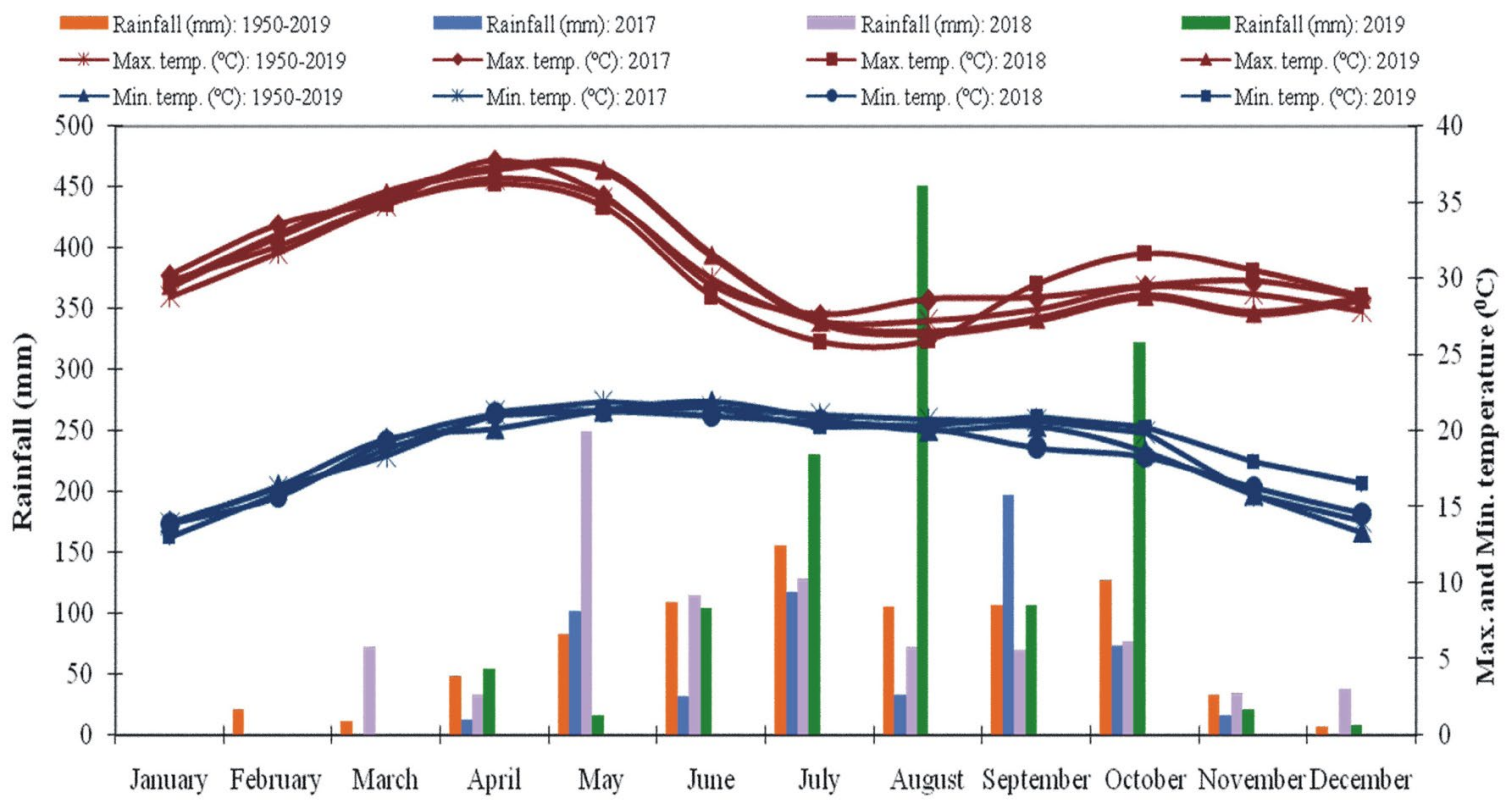
Monthly meteorological data during cropping years (2017–2019) and the average of 69 years (1950–2019) at Main Agricultural Research Station, Dharwad, India.

In comparison to the average rainfall over the previous 69 years, the three cropping years at the experimental location recorded rainfall of 582.8, 892.0, and 1316 mm in 2017, 2018, and 2019, respectively (Fig. 4). In each of the 3 years, April witnessed the highest maximum temperature (37.7, 36.2, and 37.2 °C in 2017, 2018, and 2019 respectively). Similarly, the lowest temperature was13.9, 13.9and 13.0 °C during January month of 2017, 2018 and 2019 respectively. The mean maximum relative humidity ranged from 90.9% during September 2017 to 88.0 during both July 2018 and August 2019. The minimum mean relative humidity ranged from 35.1% during February 2017 to 46 and 40% during February 2018 and 2019 respectively.

### Treatment details

The experiment was laid out in randomized block design with three replications consisted of eight treatments involving different soil and crop management practices viz*.*, Farmers’ practice (FP), ecological intensification (EI), EI minus recommended tillage management (EI-RTM), EI minus recommended nutrient management (EI-INM), EI minus recommended planting density management (EI-RPM), EI minus recommended water management (complete rainfed, EI-RIM), EI minus recommended weed management (EI-IWM) and EI minus recommended disease and insect management (EI-IPM). Each treatment was on a fixed plot for all three years. Treatment details are elaborated in Table 13.

**Table 13:** Description of agronomic practices followed for each treatment.

Treatment	Treatment details	Details
T1	Farmers’ practice (FP)	Deep ploughing during summer by a tractor followed by 2–3 passes of harrow either tractor or bullock drawn. Maize seeds drilled by bullock drawn drills manually at 60cm row to row and intra-row spacing varies from 10 to 30cm at 5-6cm depth. Seeds germinate by soil moisture retained due to preceding rains. No soil covered with crop residue may subject runoff, soil moisture loss through evaporation, top dressed nitrogen subject vitalization. Weed menace due to sufficient radiation and moisture. Farmers’ practice is devoid of recommended RTM, INM, IWM and IPM. And in place, farmers adopted practice for nutrient supply, weed and pest control were adopted
T2	Ecological intensification (EI)	EI treatment includes all best management practices (BMP) such as minimum tillage at the time of sowing, only to the extent of opening shallow furrows at a depth of 10 cm using tractor operated seed-cum-fertilizer drill and planting of maize at 60 cm × 20 cm with recommended quantity of FYM (7.5 t/ha) which was applied to field two weeks before planting. Recommended dose of fertilizers (150:65:65 kg N, P_2_O_5_ and K_2_O ha^−1^, respectively were placed in furrows opened at 5 cm away from the crop row and covered with soil. Application of bio-fortified zinc and iron each @15 kg/ha was made at the time of sowing. Soil mulching with one third of maize and chickpea crops residue @ 5.0 t/ha). Half of the required nitrogen and full doses of phosphorous and potash were placed below the seed in opened furrows at the time of sowing. The remaining nitrogen was top dressed in two equal splits, first at knee-high stage and second dose corresponding to tasseling stage. Weeds were managed by post-emergence application tembotrione 120 g a.i./ha at 25 days after sowing (DAS). Protective irrigation was provided to crop at flowering stage during first year as there was long dry spell. Subsequent two years there was sufficient soil moisture due to excess rain. Fall armyworm and Turcicum leaf blight are the major pest of maize in the tropics. Crop mmanagement operations were done as per the recommended package of practices such as RTM, RPM RIM, IWM and IPM
T3	EI-recommended tillage management (RTM)	It was exclusion of RTM and inclusion of all other EI practice. However, it included farmers’ adopted tillage having deep ploughing during summer by a tractor followed by 2–3 passes of harrow either tractor drawn, or bullock drawn. Sowing by marking the rows with a manual seed drill. Inter-cultivation at 30 and 40 DAS
T4	EI-integrated nutrient management (INM)	Exclusion of INM practice and inclusion of all other EI practices. However, it includes farmers adopted nutrient practices such as application of 1.25 kg DAP at sowing and top dressing of 300kg urea in two equal splits at 30 and 50 DAS. It accounts for 138 kg nitrogen and 42 kg phosphorus with no potassium application
T5	EI-recommended planting density management (RPM)	Exclusion of RPM and inclusion of other EI practice. farmers’ adopted planting spacing (60 cm row and intra-row is not maintained) and no thinning and gap filling practices
T6	EI-recommended irrigation management (RIM)	Exclusion of RIM and inclusion of other EI practice. farmers’ adopted soil moisture by growing under rainfed situation
T7	EI-integrated weed management (IWM)	Exclusion of IWM and inclusion of other EI practice. For weed control, one hand weeding and one inter-cultivation is practiced which is followed by farmers
T8	EI-integrated pest management (IPM)	Exclusion of IPM and inclusion of other EI practice. One spray of insecticide and fungicide was done at 30 DAS and 60 DAS for control of Fall armyworm and Turcicum leaf blight respectively

Like EI practice, all other remaining treatments were incorporated with crop residue except FP @5 t/ha.

### Cultivation method

The EI treatment comprised all best management practices (BMPs), such as mulching the soil with leftover maize and chickpea crop residue and planting maize at a specified spacing of 60 cm × 20 cm with 7.5 t/ha of FYM that was spread on the field two weeks prior to planting. In furrows that were opened at 5 cm from the crop row, the recommended fertilizer dosage of 150:65:65 kg/ha N, P_2_O, and K_2_O were applied and then covered with soil. At the time of sowing, full doses of phosphorus, potassium, and half of the recommended nitrogen were applied. The remaining quantity of the nitrogen was top-dressed in two equal portions, the first at weeding stage, which corresponds to the knee-high stage, and the second dose, which corresponds to the tasseling stage. At 25 days after sowing (DAS), tembotrione 120 g a.i./ha was applied post-emergence to control weeds. In the first year due to a prolonged dry spell, protective irrigation was given to the crop at the flowering stage. Due to an abundance of rain in the two years that followed, the soil was sufficiently moist. In the tropics, fall armyworm and turcicum leaf blight are the two main pests of maize. Apart from EI-RPM, crop management measures were performed in accordance with the prescribed set of practices to manage these pests. Succeeding chickpea crop was planted uniformly in all treatment plots and recommended fertilizer dose of 50:25:0 kg N, P_2_O_5_ and K_2_O ha^−1^, respectively was applied at the time of planting. Data were recorded on growth and yield attributes of both crops by following standard procedures. Maize farmers usually employ conventional tillage and establish maize crops manually. Irrigating, fertilizing, weeding, and harvesting, with periodic advice by researchers. Inputs such as seeds, fertilizers, tillage machinery and herbicides were applied as per the treatment requirement.

### Analysis of available nutrients in the soil

At the beginning of the experiment, the physico-chemical properties of the soil were examined (Table 14). Soil samples were collected after crop harvest and analyzed for available nitrogen, phosphorus, and potassium (kg ha^−1^) using the modified alkaline potassium permanganate method^73^, Olsen's method^74^, and neutral normal NH_4_OAc extractant method)^75^, respectively. The micronutrients, zinc and iron were extracted from soil samples using DTPA reagent and measured using an atomic absorption spectrophotometer^76^. Soil organic carbon was estimated by Walkley and Black’s method^77^.

**Table 14:** Initial physico-chemical properties of experimental soils.

Properties	Value	Method
I. Physical properties		
1. Particle size analysis	6.5	
Coarse sand (%)	15.7	International pipette method^91^
Fine sand (%)	18.1	
Silt (%)	59.3	
Clay (%)	1.32	
2. Bulk density (Mg/m^3^)	6.5	Core sampler method^92^
3. Soil moisture holding capacity (0–30 cm depth)	4.2 cm	Gravimetric method
II. Chemical properties		
Electrical conductivity (dS/m)	0.31	EC bridge^93^
pH	7.95	1: 2.5 soil: water suspension^93^
Organic carbon (%)	0.43	Walkley and Black's wet oxidation method^93^
Available N (kg/ha)	161.50	Alkaline permanganate method^94^
Available P_2_O_5_ (kg/ha)	16.90	0.5 M NaHCO_3_ extractant, Olsen's method^74^
Available K_2_O (kg/ha)	317.5	Extraction with neutral normal NH40AC^92^. Flame photometer method^95^
DTP A extractible micronutrients (ppm)		
Cu	1.18	Extraction with DTPA reagent^76^
Fe	4.06	
Mn	12.20	
Zn	0.23	

### Soil microbial enumeration

Freshly collected soil samples from each plot were analyzed for total bacterial, fungal, and actinomycetes populations using the commonly employed serial dilution plate count method with soil extract agar for bacteria^78^, Martin rose bengal agar for fungi^79^, and Kuster's agar for actinomycetes^80^. Plates were cultured in an incubator at 28 + 2 °C for 3 to 6 days before colony counts were recorded. Microbial populations were expressed as the number of colonies forming units per gram of soil or per milliliter (ml) of sewage water.

### Estimation of enzyme activity in soil

The phosphatase activity of a soil sample was measured using an established procedure^81^. One gram of soil sample was transferred in a 50 ml Erlenmeyer flask, followed by 0.2 ml toluene and four ml of modified universal buffer (pH 7.5). One milliliter of P-nitrophenol phosphate solution prepared in modified universal buffer was added to the flasks, and the contents were swirled for 2 min. The flasks were paused and incubated for one hour at 37 °C. Following incubation, one milliliter of 0.5 M CaC1_2_ and four milliliters of 0.5 M NaOH were added to the flask, stirred, and filtered using Whatman No. 42 filter paper. Using a spectrophotometer, the intensity of the yellow color obtained was examined at 420 mm in comparison to the blank reagent. Phosphatase activity in soil samples was measured in milligrams (mg) of para-nitrophenol phosphate. The activity of dehydrogenase in soil samples was evaluated as well using the standard procedure^82^. In test tubes, ten grammes of soil and 0.2 g CaCO_3_ were properly mixed and dispensed. One ml of a 3% aqueous solution of 2, 3, 5 triphenyl tetrazolium chloride (TIC) was added to each tube. One ml of 1% glucose solution and eight ml of distilled water were used to create a thin film of water over the soil layer. Rubber bungs were used for sealing the tubes, that were then incubated at 30 °C for 24 h. After incubation, the contents of the tube were washed into a small beaker, and a slurry was created by adding 10 ml methanol. Whatman No. 50 filter paper was used to filter the slurry. The soil was rinsed with methanol many times until the filtrate was red-free. In a volumetric flask, the filtrate was pooled and made up to 50 ml with methanol. Using a spectrophotometer, the intensity of the red color was determined at 485 nm against a methanol blank. The amounts of formazan in soil samples were measured by comparing them to a standard curve developed with graded formazan concentrations. The results were expressed in mg of triphenyl format.

### Turcicum leaf blight severity

The severity of turcicum leaf blight was recorded at the dough stage of crop growth (90–95 days after sowing) using modified 0–9 disease rating scale^83^ on ten randomly selected plants. A further per cent disease index was calculated using the following formula^84^.

$${\text{PDI}}=\frac{\mathrm{Sum \; of \;all \; disease \;ratings}}{\mathrm{Total \; number \; of \;plants \;observed}}\times \frac{100}{\mathrm{Maximum \; disease \; grade}}$$

### Incidence of the fall armyworm

In each treatment, ten plants were randomly selected and observations on the number of larvae per plant were recorded. Further, this data was used for calculation of mean larval population per plant by using the following formulae.

$$\mathrm{Incidence \; of \; larvae \; per \; plant}=\frac{\mathrm{Number \; of \; larva}}{\mathrm{Number \; of \; plants \;observed}}$$

### Energetic and bio-economic analysis

All the agricultural inputs such as seeds, fertilizers, labor, animals, electricity, machinery, organic manures etc*.* and all the agricultural outputs such as grain and straw have their own equivalent energy and expressed in Mega Joules (Table 15). The energy balance was calculated using the data on input energy and output energy. From these, the net energy returns, energy use efficiency, energy productivity and specific energy were calculated using the following formulae^26,85–88^.

$${\text{Net}}\;{\text{energy}}\;{\text{returns}}\left( {{\text{MJ}}/{\text{ha}}} \right) = {\text{Output}}\;{\text{energy}} - {\text{input}}\;{\text{energy}}$$

**Table 15:** Energy equivalents (MJ/unit) used for energy input and output calculations.

Sl. no.	Particulars	Energy equivalent (MJ unit^−1^)	Reference
1	Human labor (person/h)	1.96	Barut et al.^59^; Kumar et al.^69^; Shahin et al.^60^; Yadav et al.^96^
2	Machinery/h	64.80	
3	Diesel/l	56.31	
4	FYM/ ton	303.1	Avval Mousavi et al.^97^
5	Nitrogen (N)/kg	66.14	Barut et al.^59^; Kumar et al.^69^; Rahman and Rahman^98^; Shahin et al.^60^; Shahan et al.^99^
6	Phosphorus (P_2_O_5_)/kg	12.44	
7	Potassium (K_2_O)/kg	11.15	
8	Zinc sulphate/kg	20.90	Nassiri and Singh^89^
9	Herbicides and insecticides/kg	120.0	Kumar et al.^69^; Shahin et al.^60^; Shahan et al.^99^
10	Irrigation mm/ha	1430.6	Barut et al.^59^; Kumar et al.^69^; Rahman and Rahman^98^; Shahin et al.^60^
11	Maize seeds/kg	15.20	Rahman and Rahman^98^; Yadav et al.^96^
12	Maize grain/kg	14.70	Barut et al.^59^; Kumar et al.^69^; Rahman and Rahman^98^; Shahin et al.^60^; Yadav et al.^96^
13	Maize stover/ton	18.0	Barut et al.^59^; Kumar et al.^69^; Rahman and Rahman^98^; Yadav et al.^96^
14	Chickpea seeds/kg	25.0	Thyagaraj^100^
15	Chickpea grains/kg	25.0	
16	Chickpea haulm/ton	1000	

Where multiple references are shown, the average was calculated.

$$\mathrm{Energy \;use \; efficiency}=\mathrm{Output \;energy}({\text{MJ}}/\mathrm{ha })/\mathrm{Input \; energy}({\text{MJ}}/\mathrm{ha })$$

$$\mathrm{Energy \; productivity }\left(\mathrm{kg \; MJ}/{\text{ha}}\right)= \frac{\mathrm{Output \; energy }({\text{MJ}}/\mathrm{ha })}{\mathrm{Input\; energy }({\text{MJ}}/\mathrm{ha })}$$

$$\mathrm{Specific \; energy }\left({\text{MJ}}/\mathrm{kg }\right)=\frac{\mathrm{Output \;energy }({\text{MJ}}/\mathrm{ha })}{\mathrm{Input \;energy }({\text{MJ}}/\mathrm{ha })}$$

Maize equivalent yield (MEY) was calculated by considering prevailing market prices of two crops and expressed in q ha^−1^.

$$\begin{aligned} {\text{MEY for intercrop }} & = {\text{ Maize yield }} + [\{ {\text{chickpeayield }}({\text{q}}/{\text{ha}}) \, \hfill \\ & \quad \times {\text{Price of chickpea}}({\text{Rs}}.{\text{ q}}^{{ - {1}}} )\} \, \div {\text{Maize price }}({\text{Rs}}.{\text{ q}}^{{ - {1}}} )] \hfill \\ \end{aligned}$$

Prices for inputs and outputs for each season were monitored by local markets. Fuel use for land preparation, planting, and irrigation was quantified^31^. This information was used for partial budgeting and was converted to megajoule (Mj) equivalents for energetic analysis^31,89^. Net income was determined by subtracting all variable costs from the gross returns from grain and exported stover or straw. Energy inputs and outputs were computed for recycled grain and stover, as well as total biomass exported. Energy use efficiency (EUE) was calculated by dividing total output of energy for rice and maize (Mj/ha) by total inputs of energy (Mj/unit). Specific energy (SPE) was estimated by dividing total energy inputs (Mj/inputs) by grain + stover yield (kg/ha). Subsequently, energy productivity (EP) was derived by dividing grain yield (kg/ha) by total energy input (Mj/ha).

The price of the inputs in rupees (Rs.) at the time of use was considered to figure out the cost of cultivation per hectare treatment wise and expressed in Rs./ha. For working out the cost of inputs, land preparation, inter-cultivation, all applied fertilizers, FYM, seed, plant protection chemicals, irrigation, men, and women wages from planting through harvesting, drying, processing, and marketing of produce were all included. A gross return per hectare was computed by taking into consideration the market price of the commodity after harvest and the yield of grain per hectare and was expressed in Rs. per hectare. The net return per hectare was calculated treatment wise by subtracting the total cost of cultivation from gross return and expressed in Rs./ha.

$${\text{Net return }}\left( {{\text{Rs}}./{\text{ha}}} \right) \, = {\text{ Gross return }}\left( {{\text{Rs}}./{\text{ha}}} \right) \, {-}{\text{ Cost of cultivation }}\left( {{\text{Rs}}./{\text{ha}}} \right)$$

The benefit cost ratio was calculated as follows.

$$\mathrm{Benefit \; cost \;ratio }\left({\text{B}}{-}{\text{C}}\right)=\frac{\mathrm{Gross \;retun }({\text{Rs}}./{\text{ha}})}{\mathrm{Cost \;of \;cultivation }({\text{Rs}}./{\text{ha}})}$$

### Weed dynamics

Total weed population/m and total weed dry matter was recorded at 30 and 60 DAS under each treatment in 0.5 m^−2^ quadrat. Data on weed density and weed biomass were transformed using square root transformation.

### Statistical analysis and the interpretation of data

The data collected from various parameters on soil and plant growth, biochemical, biophysical and yield attributes from field experiment at different growth stages were subjected to statistical analysis^90^. These data were subjected to ANOVA (analysis of variance) in accordance with field design (Randomized Complete Block Design) using M-Stat package to quantify and evaluate the sources of variation. The level of significance used in ‘F’ and ‘T’ test was P = 0.05. Critical difference (LSD) values were calculated whenever the ‘F’ test was found significant.

## Data Availability

All data generated or analyzed during this study are included in this publication article.
